# HPCache: memory-efficient OLAP through proportional caching revisited

**DOI:** 10.1007/s00778-023-00828-7

**Published:** 2023-12-22

**Authors:** Hamish Nicholson, Periklis Chrysogelos, Anastasia Ailamaki

**Affiliations:** 1 EPFL, Lausanne, Switzerland; 2 Oracle, Zurich, Switzerland; 3 Google, Zurich, Switzerland

**Keywords:** Analytical query processing, Storage engines, Storage-resident data, NVMe, High-bandwidth storage

## Abstract

Analytical engines rely on in-memory data caching to avoid storage accesses and provide timely responses by keeping the most frequently accessed data in memory. Purely frequency- and time-based caching decisions, however, are a proxy of the expected query execution speedup only when storage accesses are significantly slower than in-memory query processing. On the other hand, fast storage offers loading times that approach fully in-memory query response times, rendering purely frequency-based statistics incapable of capturing the impact of a caching decision on query execution. For example, caching the input of a frequent query that spends most of its time processing joins is less beneficial than caching a page for a slightly less frequent but scan-heavy query. Thus, existing caching policies waste valuable memory space to cache input data that offer little-to-no acceleration for analytics. This paper proposes HPCache, a buffer management policy that enables fast analytics on high-bandwidth storage by efficiently using the available in-memory space. HPCache caches data based on the speedup potential instead of relying on frequency-based statistics. We show that, with fast storage, the benefit of in-memory caching varies significantly across queries; therefore, we quantify the efficiency of caching decisions and formulate an optimization problem. We implement HPCache in Proteus and show that (i) estimating speedup potential improves memory space utilization, and (ii) simple runtime statistics suffice to infer speedup. We show that HPCache achieves up to a 1.75x speed-up over frequency-based caching policies by caching column proportions and automatically tuning them. Overall, HPCache enables efficient use of the in-memory space for input caching in the presence of fast storage, without requiring workload predictions.

## Introduction

Improvements in CPU and DRAM efficiency allow analytical engines to place frequently accesseddatasets in-memory [41]—avoiding slow storage accesses^1^. However, CPU and DRAM improvement rates have slowed in recent years, while advances in flash storage have enabled increased persistent-storage bandwidth [18, 26, 27, 36]. As a result, storing the working set in memory is no longer always advantageous. For example, when a query is CPU or memory latency bound, it has lower throughput than storage bandwidth, so storing the query’s entire input in memory is wasteful because it is possible to achieve the same execution time with the input located on storage. Instead, it would be more beneficial to use the same memory for another query.

**Fig. 1:**
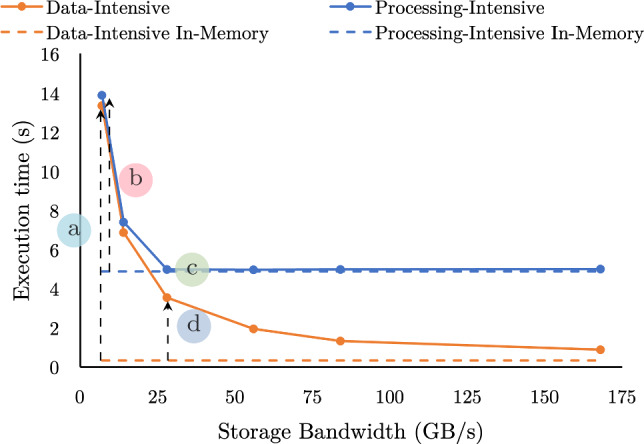
Execution time of two queries over NVMe resident data as the storage bandwidth increases compared with in-memory execution

For decades, database designs were based on the assumption that storage IO is the bottleneck in execution times and relied on in-memory caching in the buffer pool to bypass it. There are two lines of work that improve buffer pool performance for analytics: (i) improving the efficiency of accessing buffer pool pages [25, 29] and (ii) improving the probability that frequently used pages remain in memory [11, 12, 22, 31, 34, 35, 38]. In the first line of work, fast access to the buffer pool accelerates storage [3, 25, 29] and reduces the buffer pool overhead but relies on the effectiveness of the buffer pool policy to accelerate query execution. In the second line of work, frequency-based eviction policies improve the cache hit rate. High-bandwidth storage, like multiple NVMes per machine, however, allows for data loading times that are competitive to in-memory query processing (Fig. 1). Thus, improving the hit rate no longer implies faster analytics, and as a result, the available in-memory space is underutilized, slowing down query execution.

In this paper, we propose HPCache, an eviction policy and tuning agent that optimizes the caching efficiency of the buffer pool for analytical workloads on high-bandwidth storage. We show that (i) caching pages that have the same access frequency can yield variable query acceleration results, and (ii) efficient cache use should aim for partial column caching to avoid diminishing returns. HPCache is a buffer management policy that considers both the query frequency and the acceleration impact of any caching decision to derive a memory-efficient caching decision. Toward that end, HPCache (i) examines the query execution to understand caching benefits and (ii) automatically tunes the caching priority and in-memory column space budget based on past execution behavior. We extend the previous version [30] of HPCache to (1) reduce artifacts resulting from looking back a fixed number of queries during decision-making and (2) enable custom policies. Further, we provide a design that allows simple integration of HPCache with an existing analytical engine. We revisit the sensitivity analysis of HPCache using a hardware configuration with sufficiently higher storage bandwidth. Overall, HPCache improves the efficiency of in-memory data caching for analytics, allowing faster query execution for a given memory budget.

In summary, HPCache makes the following **contributions**:

- We show that in the presence of high bandwidth storage, frequency-based in-memory caching policies cache inputs that provide little query acceleration (Sect. 2).
- We propose HPCache, a policy that enables efficient memory utilization by considering the expected query acceleration of different caching decisions (Sect. 3). To avoid unreliable predictions, HPCache continuously tunes the caching policy based on run-time statistics (Sect. 4).
- We extend HPCache to support varying query importance and non-fixed query window lengths (Sect. 4.4) and describe HPCache’s integration with the rest of the execution engine (Sects. 5, 6).
- We provide an extensive evaluation of HPCache to show how proportional caching improved query response times by up to 1.75x for both small and large cache capacities (Sect. 7).

Overall, HPCache enables analytical query processing to efficiently use the available in-memory space when accessing larger-than-memory datasets, allowing faster analytics for the same memory budget, or decreased memory footprint without execution time degradation.

The additional content compared to the earlier publication of this work [30] is mainly discussed in Sects. 4.4, 5, 6, and through new experiments in Sect. 7.

## In-memory and high-bandwidth Storage

Directly attached NVMe arrays have enough bandwidth to invalidate the general rule of thumb that scanning persistent data is always slower than in-memory execution. The rest of this section shows how high read bandwidth affects the execution speedups achieved by in-memory caching and how frequency-based caching policies result in ineffective eviction decisions. Finally, we quantify the relative value of caching different data.

Fast storage While local storage was once considered slow relative to memory, recent advances in flash technology have resulted in servers having comparable storage read bandwidth and memory bandwidth. Recent NVMes can sustain GB/s of read bandwidth, e.g., an Intel D7-P5600 achieves 7 GB/s. Furthermore, CPUs support 100 s of PCIe 4.0 lanes per socket [15], which allows packing multiple NVMes on the same server to achieve 10 s-100 s GB/s of aggregated read bandwidth—just a single order of magnitude lower than the CPU memory bandwidth.

*Quantifying “fast”* Still, an order of magnitude more bandwidth is significant. Thus, analytical engines rely on CPU memory for a wide range of operations. In addition to higher read bandwidth, CPU memory also provides better random IO performance and lower latency than NVMe arrays. As a result, CPU memory has been a crucial element in enabling efficient in-memory joins [5, 6, 8] and analytics in general [9]. Thus, for many queries, reading the input data is only a small portion of the memory operations and only dominant for scan-heavy and few-small-join queries [37].

Performing random or latency-sensitive data accesses directly on NVMe-resident data would impose a significant overhead compared to accessing the same data from memory. In contrast, sequentially accessing data from NVMe arrays rather than from memory can have a minimal impact on response times ; especially when random and latency-sensitive accesses or even compute costs dominate the execution time of complex queries. As such, when query complexity increases, and the sequentially accessed input data remains the same, the impact of slower sequential access, e.g., from a slower medium, reduces and can often be overlapped with the rest of the execution. This shrinking of the relative contribution of sequential accesses to the total execution time as query complexity increases, combined with high NVMe storage bandwidth, allows for sequentially scanning data from the NVMe-array storage with a minor penalty—a reverse application of Amdahl’s law. Instead, the caching layer can use the saved space for simpler, sequentially scan-heavy queries.

To quantify the aforementioned query complexity, we define the *processing intensity* of a query: an indicator for the insensitivity of a query to accessing its inputs from storage rather than from memory. Specifically, we define the processing intensity of a query as the ratio between its in-memory execution time and the time to scan its inputs from memory. For example, with a memory bandwidth of 100 GB/s and 10 GB query input data, a query that executes in 20 s would have a processing intensity of 200. With the same setup, a query that executes in 1 s would have a processing intensity of 10. A *processing-intensive query* has a relatively high processing intensity and is relatively insensitive to whether its input data are in memory. The converse is a *data-intensive query*, which has a low processing intensity and is largely bottlenecked by scan throughput.

**Fig. 2:**
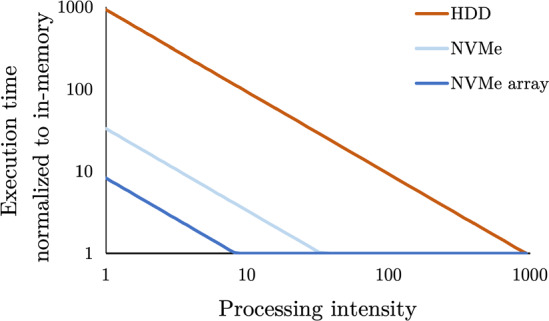
Conceptual slowdown relative to fully in-memory processing for queries with increasing processing intensity. Modeled as a simplified, without caching, version of the model introduced in Sect. 4, assuming that an HDD, an NVMe drive, and an array of 4 NVMe drives are, 0.1%, 3% and 13% of memory bandwidth, respectively

What is a low or high processing intensity, and consequently, the slowdown of accessing sequential inputs from storage relative to memory is a function of storage bandwidth. Figure 2 models the execution time of increasingly processing-intensive queries normalized to the in-memory execution time. Each of the plotted lines shows the slowdown relative to fully in-memory execution. The sloped component of each line shows the range of processing intensities for which queries are bottlenecked by scanning inputs from the respective media. The ranges where the lines flatten, approaching in-memory query processing speeds, illustrate when the query execution is bottlenecked by in-memory processing and not scans. As storage bandwidth increases, a greater range of queries can run with fully in-memory performance while keeping their inputs entirely in storage. For example, in Fig. 2, with a single HDD, only queries with a processing intensity of over nearly 1000 are not bottlenecked by the throughput of the scan of HDD-resident input data. In contrast, the transition point from scan throughput bound to processing bound is at a processing intensity of just over 32 and 8 for a single NVMe drive and an array of NVMe drives, respectively.

In-memory analytics vs. data loading: a race Contrary to common belief, storage accesses no longer have a catastrophic impact on analytical query response time. We show this concretely using queries from the Star Schema Benchmark (SSB) [32]. Fig. 1 shows the execution time for a data-intensive (SSB Q1.3) and processing-intensive query (SSB Q3.1) as the storage bandwidth increases. Each has the same working set size of 96 GB. The storage bandwidth is controlled by varying the number of drives the data are striped across. Both queries access 96 GB of input data, but the data-intensive query has one cache-resident join while the processing-intensive query has four higher-cardinality joins. With 7 GB/s of storage bandwidth, both queries are bottlenecked by reading data from the NVMe storage. The data-intensive and processing-intensive queries are 41.2x () and 2.8x () slower than in-memory execution, respectively; the processing-intensive query is marginally slower as it first builds the hash tables for its hash joins. However, with 28 GB/s, the processing-intensive query is just 1% slower than in-memory (), as the storage bandwidth saturates the query’s processing throughput. Though, the data-intensive query is still 10.9x slower (). Faster storage has diminishing returns for extremely data-intensive queries, as both query processing and data loading compete for the available memory bandwidth. Caching the processing-intensive query’s input data has minimal speedup potential with storage bandwidth in excess of 28 GB/s: the query, either way, spends most of its time processing the joins. In contrast, the data-intensive query will benefit from caching for all storage bandwidths, though the time savings from caching depends on the storage bandwidth. Overall, whether in-memory caching of the input data will reduce query response time or not is query-dependent, even for simple queries.

Memory efficiency of caching While minimizing storage accesses does not harm single-query execution, it leads to inefficient memory use when considering multiple queries. With data loading times comparable to execution times for some queries, caching input data for a query that spends most of its time on non-input operations can result in wasting memory that could be used to accelerate another query. Further, it contradicts the prior wisdom of caching the most frequently accessed data. The most common caching heuristics for analytics involve prioritizing data that has not been used for a long time (LRU [40]), that was recently consumed (MRU [12]), or a combination thereof and second-chance approaches [22]. However, even if Q3.1 of the previous example were executed significantly more frequently than Q1.3, caching its input would provide little benefit: caching reduces storage IO, but as shown above, Q3.1’s execution time will improve very little. In contrast, caching the inputs of Q1.3 will accelerate each execution of Q1.3. Thus, treating all IO savings equally results in suboptimal query response times.

## HPCache: hybrid proportional caching

Based on the above observations, we propose Hybrid Proportional Caching (HPCache), a new data placement strategy that makes caching decisions based on the expected query response time reductions. Rather than aiming to reduce storage accesses, HPCache builds on three key principles to efficiently use the in-memory space: (i) not all inputs provide the same query acceleration, (ii) pages should be prioritized based on the expected impact on query execution time, (iii) optimal caching decisions should aim for the sweet spot of matching query execution times with loading the remaining data. The rest of this section outlines the above key principles and their effect on in-memory caching decisions.

**Not all bytes are the same.** Some queries are more sensitive to the location of their input data than others. Multiple factors affect the query response time, including the query access patterns, the data placement of both intermediate and input data, and the ability of the execution strategy to effectively utilize the available hardware resources, e.g., through prefetching, vectorization, and cache-awareness. Considering the query execution time, we can roughly classify queries into two broad categories: data-intensive queries whose execution time is sensitive to the bandwidth available to access input data, and processing-intensive queries that spend most of their time accessing intermediate structures like join hash tables.

Caching different inputs in memory provides different response time gains. *Thus, we use the expected benefit (Sect.* 4.1*) when deciding between alternative caching decisions to use the in-memory space efficiently. *

**Fig. 3:**
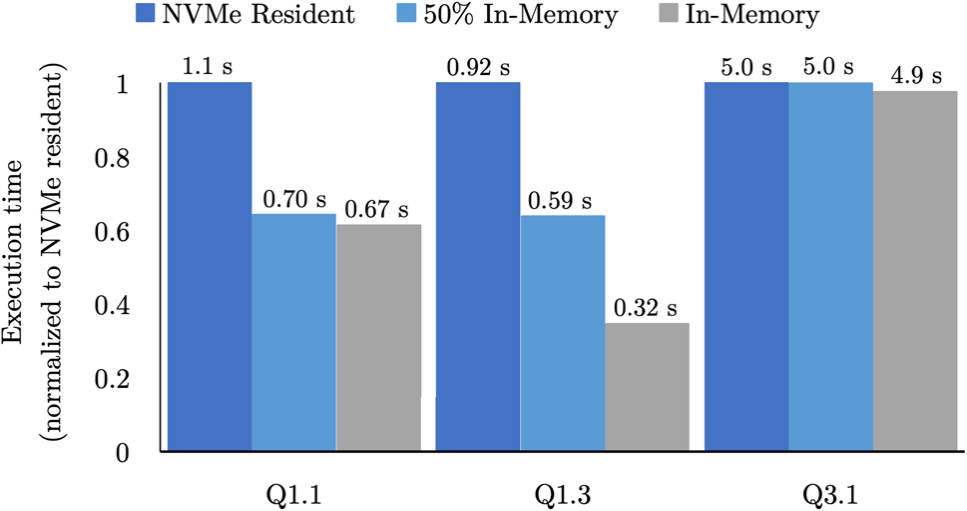
Normalized execution time for three different data placements using 24 NVMe drives with 168 GB/s read bandwidth. The numbers above the bars show the absolute execution time in seconds

**Impact- and frequency-based caching.** The importance of caching the input of a query, and that’s the caching efficiency it observes, depends on its processing intensity: a data-intensive query with low processing intensity will observe a higher (relative) speedup than a processing-intensive query when its inputs are cached in memory.

Figure 3 shows the execution time of three analytical queries, from the Star Schema Benchmark (SSB) [32] at scale factor 1000, when using three data placement methods: fully in-memory, fully NVMe resident, and a hybrid configuration with half the input data in-memory and half NVMe resident. Both Q1.1 and Q1.3 are data-intensive queries, and thus, their execution time is nearly halved when moving half of their inputs in memory. However, when moving to fully in-memory, the two queries have different execution times despite their equal input size due to their different processing throughputs; Q1.3 has a higher processing throughput, equivalently a lower processing intensity, and thus benefits more by having all of its inputs in memory. Q3.1 is a processing-intensive query, so it executes in nearly the same amount of time regardless of the location of its inputs. Allocating memory equally to cache the inputs of these three queries would result in a slower speed up than preferentially caching the inputs of the data-intensive queries.

However, per-query caching efficiency is not the only metric that matters when selecting which query inputs to cache: query frequency can also boost or reduce the overall efficiency. In a given workload, if the same or similar queries appear many times, then there is a multiplicative benefit to accelerating the repetitive query by caching its inputs.

To optimize caching efficiency, HPCache combines traditional frequency-based approaches with impact-based query acceleration expectations to achieve the best of both worlds. Specifically, it weights queries and input columns based on their occurrence frequency but calculates the overall expected execution time instead of purely optimizing the number of storage accesses. *We model query execution as a flow (Sect.* 4.2*) to approximate how different data placements would affect the total execution time for a sequence of queries and select the best placement given a memory budget.*

**Fig. 4:**
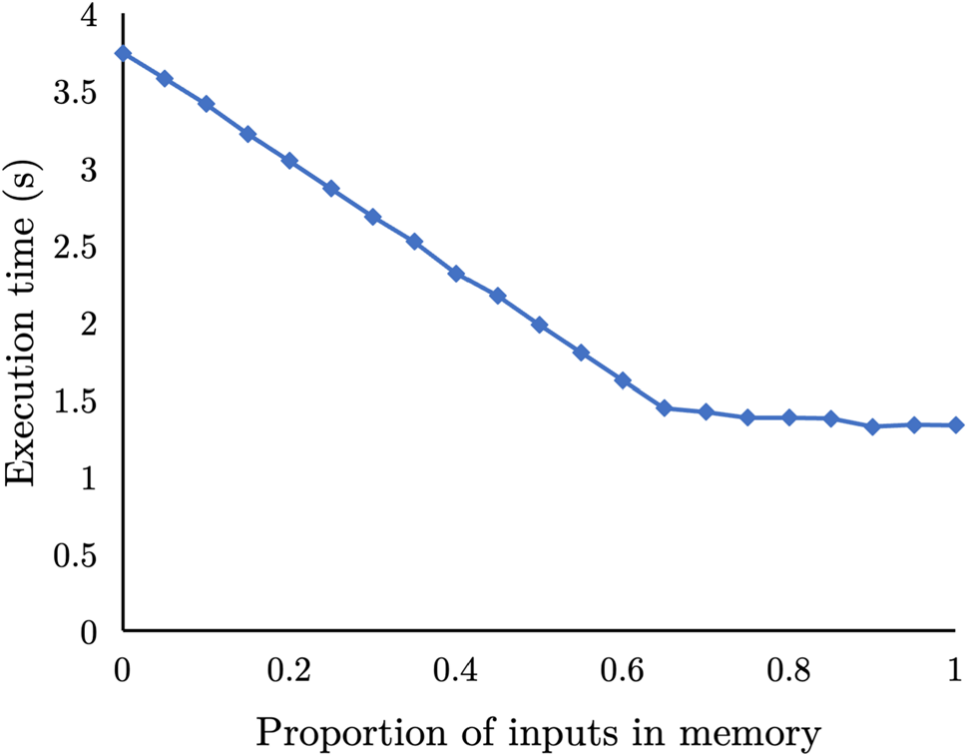
Execution time for SSB Q2.2 with increasing proportions of input data cached in memory (Four NVMes-28 GB/s storage bandwidth)

**Partial input caching.** Caching entire columns is wasteful: even in the simplified case of a single query, caching more than a specific fraction of a column provides minimal additional query speedup. Figure 4 shows how the execution time of SSB Q2.2 improves as we increase the percentage of in-memory input data while the rest resides on an NVMe. Initially, the execution is dominated by the data loading time and thus improves linearly as more data is found in the in-memory cache. However, despite using more memory, the query execution time sees almost no reduction after a specific point. From that point up to having fully in-memory inputs, query execution is dominated by the actual processing. Thus, additional caching has no benefit: it would be preferable to use it for another query. HPCache limits the number of pages that are cached for each column to avoid diminishing returns from caching unnecessarily high portions of a specific column. *The limit is determined in a per-column base, and it is proportional to the expected impact of caching the corresponding column (Sect.* 4.3*). *

## Tuning and monitoring

To materialize HPCache, we provide a model that captures per-input caching impact (Sect. 4.1) and use it to provide a memory-efficient caching configuration (Sect. 4.2). Finally, we show how HPCache continuously adapts its caching configuration based on updated estimates and caching configurations (Sect. 4.3).

### Impact modeling

We model the impact following a two-step procedure: (i) model the impact of caching the inputs of a specific query and (ii) combine impacts from multiple queries to determine the overall impact of caching a column.

**The benefit of caching a query input.** To model the impact of caching a specific query input, we model the execution time for the pipeline [28] consuming the corresponding input^2^: by definition, caching the input will only change the performance of that pipeline. Any other pipeline that joins with the current one will use a newly materialized data structure produced by the modeled one. Further, the pipeline operates like a pipe as it consumes its input at a specific rate. This rate is limited either by input access bandwidth or the pipeline’s maximum throughput. Thus, if we have the size of the pipeline inputs (*B*), the storage bandwidth ($S_{bw}$), the memory bandwidth ($M_{bw}$), the proportion of inputs in memory (*x*), as well as the pipelines maximum throughput ($P_{bw}$), then we can approximate the execution time for partially cached inputs as

1 $$\begin{aligned} T_{pipeline{}, x\%} = \max \left( \frac{(1-x)*B}{S_{bw}}, \frac{x * B}{M_{bw}}, \frac{B}{P_{bw}}\right) \end{aligned}$$

This results in a line similar to Fig. 4 as the proportion of inputs in memory (*x*^3^), varies from 0% to 100%. The query’s execution time is initially bottlenecked by storage IO (the left side of the $\max$) until the query execution time reaches in-memory processing speeds (equal first and the last terms of the $\max$). At this point, caching any additional inputs in memory does not improve the execution time. Calculating $P_{bw}$ is generally non-trivial and would require an accurate model and/or observing previous executions of similar queries. We avoid this complexity by inferring its value by inspecting, during the actual execution, the query’s processing throughput on the first few row groups and continuously updating this estimate (more details in Sect. 4.3). To calculate the reduction of execution time from one input caching rate ($x\%$) versus another ($y\%$), it is sufficient to subtract the $T_{pipeline{},x\%}$ from $T_{pipeline{},y\%}$.

**The benefit of a byte.** Some columns may be used by multiple queries. To compute the caching impact of a column, we need to aggregate the execution speed-up it will allow across multiple queries. However, as the column participates in multiple pipelines, even if it is fully in-memory, it may have to wait for other columns of the same pipeline to be loaded and vice-versa. Thus, to allow estimating the impact, we rely on a finer-grained granularity that builds the end-to-end execution time model based on per-column-per-pipeline time estimates. To build the time estimates, we need to subdivide the time estimate of the previous paragraph to column granularity. We approximate this division by splitting $T_{pipeline{},mem}$ across the columns based on their relative sizes.

### A balanced model

To provide a memory-efficient caching configuration, we model the expected execution time and the expected memory budget and formulate two optimization problems—one to optimize for the execution time given a memory footprint and one to optimize the memory footprint given a slowdown budget. Our description focuses on the former, but the same principles apply to the latter.

**Modeling as a flow.** To decouple modeling from hyperparameter tuning, such as retrieving $T_{pipeline{},mem}$ and whether future or past queries are available, we generalize the problem formulations and model execution of *L* queries, which can be either future or past ones—and we optimize execution across these *L* queries.

To calculate the total execution time (*T*) over these *L* queries, we split the queries into pipelines, and for each pipeline, we sum the time for the various participating columns. The summation across pipelines is supported by the fact that pipelines execute one after the other, while the summation across columns is valid because the accredited per-column times already split the execution time based on column size. Similarly, based on the caching ratios, we can compute the in-memory space $C\left( x\%\right)$ of having the corresponding proportions cached in memory.

For example, for two queries $Q_A$ and $Q_B$, that have a single pipeline each and they touch columns $\{i,j\}$ and $\{j, k\}$, respectively, we approximate the total execution time as:

2 $$\begin{aligned} \begin{aligned} T_{x_{i,j,k}\%}&= max\left( T^{i}_{pipeline{}_A, x_i\%}, T^{j}_{pipeline{}_A, x_j\%}\right) \\&\quad +max\left( T^{j}_{pipeline{}_B, x_j\%}, T^{k}_{pipeline{}_B, x_k\%}\right) \end{aligned} \end{aligned}$$

by making the approximation that:

3 $$\begin{aligned} T_{pipeline{}_A, x_{i,j}\%} = max\left( T^{i}_{pipeline{}_A, x_i\%}, T^{j}_{pipeline{}_A, x_j\%}\right) \end{aligned}$$

because a pipeline’s throughput will be bottlenecked by the column with the lowest available access bandwidth. For example, if the query is IO bound and column *i* is storage resident while *j* is in memory, the query will process only as fast as column *j* can be read from storage. Note that shared columns share cache proportions (e.g., $x_b\%$). Also, while the methodology could model cold caches, this formulation is optimized for query repetitions and thus ignores cold cache cases such as column *b* being fully storage resident before $Q_A$, but brought into the cache before $Q_B$ starts.

The above formulation of $T_{x\%}$ allows for capturing (i) the impact of different configurations, (ii) the shared caching proportions across queries, and (iii) the frequency-based importance of each query, as queries that repeat multiple times in the *L* window will appear multiple times in the summation.

Static tuning and optimization To provide an efficient configuration, HPCache sets up and solves a minimization problem that finds the $x\%$ that minimizes $T_{x\%}$, subject to $C\left( x\%\right) < B$, where *B* is the memory budget. We solve the optimization problem using a convex optimizer. The optimal configuration $x\%$ is then provided to the buffer pool. The buffer pool preferentially caches pages to attempt to maintain these proportions of the columns in memory using a soft-pinning mechanism (Sect. 5.3). The execution engine requests data pages from the buffer pool as it would do for any other caching policy.

### Continuous tuning

HPCache tracks query execution as it evolves, continuously adapting the caching configuration by taking into consideration the recent query and performance history. Furthermore, HPCache continuously monitors the query execution to tune its estimates of the inferred $P_{bw}$ for the currently executing pipeline.

**Looking back.** The above model optimizes the execution across *L* queries. This allows HPCache to optimize for future as well as past queries. In the default case, though, HPCache uses $L-1$ past queries combined with the current query to decide a new caching configuration. For the past queries, it estimates $P_{bw}$ based on the pipeline execution times. However, HPCache does not fix the caching ratios of the previous queries, to allow re-optimizing and reducing or increasing them based on the queries—essentially optimized under the assumption that a similar pattern as the last $L-1$ queries will repeat next.

**Monitoring and inspection.** In general, it is hard to predict $P_{bw}$ reliably for each pipeline. Instead, HPCache estimates $P_{bw}$ during query execution. In each pipeline invocation, the pipelines of interest receive a set of input blocks corresponding with one block per accessed attribute. The execution engine requests from the storage layer the pages needed for each pipeline invocation. HPCache intercepts these requests to calculate an estimate of $P_{bw}$ from the pages per second that the pipeline is processing and the known input size of the pipeline. Since many instances of the same pipeline run in parallel, we assume that pipeline processing time is equally distributed across threads.

HPCache uses the estimate of $P_{bw}$ along with the execution times of the previous queries as inputs for the optimization problem formulated in Sect. 4.2. HPCache re-solves the problem periodically in a background thread using the latest statistics, both refining its estimated $P_{bw}$ for the current pipeline and the overall optimal column proportions. The new optimal configuration is continuously supplied to the buffer pool as a maximum number of pages to cache for each column. If some columns exceed their maximum allocations in the new configuration, then the buffer manager moves pages from the over-represented column into a (logical) global free list by removing soft-pins (Sect. 5.3). Free list pages are evicted first to make room for new pages. While pages are in the free list, they are still available to threads requesting them until they are evicted.

### Proportional caching: avoiding horizon effects

Using a fixed number of queries to make caching decisions is prone to issues arising from patterns with a higher repetition period than the current window. Further, using a very big look-back query window *L*, slows down the adaptation of the caching method to new query patterns.

To reduce the impact of the query window on HPCache, we use HPCache’s flexible optimization goal. Specifically, instead of considering exactly *L* queries and giving them the same importance, we replace the optimization goal with a function that weights differently each of the past (potentially more than *L*) queries.

We set the weights using an exponential function, loosely mimicking an *exponential smoothing* process [1]. Queries that are further back in time get an exponentially smaller weight, while the currently running query gets a weight of 1. Specifically, the minimization problem becomes:

4 $$\begin{aligned} \begin{aligned} \min _{x\%}&\quad \sum _i \left( 1 - a\right) ^i T_{Q_i,x\%} \\ \text {s.t.}&\quad C_{x\%} < B \end{aligned} \end{aligned}$$

where *i* is how far ago $Q_i$ appeared, with $i=0$ for the current query, $i=1$ for the previous one, etc. $T_{Q_i,x\%}$ is the expected execution time for query $Q_i$, under caching proportions $x\%$. Parameter $a \in [0,1)$ is a smoothing factor, specifying how aggressively HPCache should adapt to new queries (as *a* approaches 1). A value of $a=0$ means that all seen queries are equally important, while a value of $a = 1$ would make HPCache consider only the current query (sensitivity analysis in Sect. 7.1).

Depending on the value of *a* some queries may have minimal impact on the final configuration and, yet, contribute a significant number of terms in the constraints and minimization goal—causing a disproportional minimization overhead versus their importance. To avoid this disproportionality, we prune the terms corresponding to queries with a factor below a threshold.

**Extensions.** Transformations of the minimization problem allow HPCache to cover various use cases. For example, increasing the weight of some queries results in creating “high-priority” queries whose acceleration is prioritized over the rest. Similarly, replacing the minimized function with

5 $$\begin{aligned} max\left( max\left( T_{Q_i,x\%}, T_{thresh}\right) \right) \end{aligned}$$

makes HPCache generate caching proportions that aim at keeping the query execution times below a response time $T_{thresh}$. Adding on that formula a scaled-down version of Eq. 4 allows the aforementioned configuration to handle queries with that, even if everything is in-memory, are slower than the target $T_{thresh}$. Overall, HPCache’s approach of revisiting the caching policy as a mathematical minimization problem and combining it with the throughput-oriented view of query execution enables new customizations of the caching process.

## Integration and interoperability

While in the previous sections, we focused on HPCache’s control logic, HPCache also needs to integrate with a variety of different execution modules. The rest of this section details how HPCache interacts with the buffer cache to maintain the appropriate data proportions and with the execution engine to retrieve the necessary information to drive both the decision-making process as well as the actual data fetching.

### Architecture overview

HPCache intercepts the calls between the analytical engine and the storage layer to retrieve the performance indicators it requires in a transparent way. Specifically, during query execution, the analytical engine requests data pages from the storage layer. HPCache intercepts these requests and uses their timings to compute three indicators that are necessary for the tuning method described in Sect. 4.3.

The first indicator is about which attribute, and row groups were requested. This allows for maintaining statistics that are required for building the capacity constraints involved in the optimization column: which columns are candidates for caching and their size. Second, it associates the above statistics with a query and operator id. This allows requests to be linked into the “impact” of optimizing each column. Third, it keeps a timeline of the requests to calculate the request interarrivals for each input column and query. These interarrivals are then used to extract the throughput corresponding to each query operator.

### Integration with the execution engine

To request a page, operators first invoke willNeedPage to mark that a data page will be needed and to initiate the page request. When the page is actually needed, the execution engine calls getPage to retrieve the actual page – blocking if the page is still not available. In addition to the page ID, willNeedPage also takes the operator ID as an argument so that the caching policy can track request origins and infer the processing throughput of the requesting operator.

The interface between HPCache and the query execution engine is asynchronous. Splitting the interface into willNeedPage and getPage allows HPCache to trigger multiple concurrent page requests and only block when enough on-the-fly requests have been issued. This is a key requirement for our throughput-oriented design and especially for hiding the latency of page fetches by overlapping it with upcoming data requests.

For example, the query executor may request six pages through willNeedPage. Each of these calls would result in HPCache calling into the storage manager to asynchronously load the corresponding page to memory if it’s not already present. However, willNeedPage does not wait for the transfer to complete, and control immediately returns to the query executor. To wait for the page transfer to complete, the executor calls into getPage. This two-step process means that while the executor waits for the first requested page, it can issue the next five requests to hide their latency.

### Integration with buffer cache

Managing caching proportions HPCache manages the data proportions in the buffer cache using a soft-pinning mechanism. When a new page is inserted into the buffer cache, the buffer cache makes a call back to HPCache, which sets a soft-pin bit in the page with a probability equal to the recommended column proportion. For example, if column B has 10 pages and a recommended proportion of 70%, then over time (ignoring evictions) column B will have, on average, 7 soft-pinned pages. HPCache determines which column a page belongs to based on the page’s ID. A soft-pin indicates that when the buffer cache needs to evict a page, it will prioritize evicting pages without a soft-pin. Soft-pins are orthogonal to traditional pins, meaning that a soft-pinned page may be evicted if it’s not pinned, while a pinned page that is not soft-pinned is not an eviction candidate. As a result, during eviction, the buffer cache prioritizes the eviction of pages with neither a soft nor hard pin (the logical free list), followed by pages with a soft-pin but no hard pin, and, as traditional buffer caches, it never evicts (hard) pinned pages. Our design traverses the page frames to find an appropriate page to evict.

Soft-pins are removed by HPCache in a background thread. This background thread periodically (every 200 ms) invokes the optimization problem to update the recommended proportions. This keeps the optimization problem off of the hot path of page requests. When the recommended proportion of a column decreases, each soft-pinned page of the column has its soft-pin removed with a random uniform probability equal to $1- \frac{recommended\ proportion}{current\ proportion}$. HPCache traverses all of the buffer frames, uses the buffer frame metadata to check which column the page belongs to, and removes soft-pins with the above probability, avoiding the use of secondary data structures to locate all of the cached pages for a particular column. A uniform random distribution of pages in memory is preferable for bandwidth-sensitive workloads, as it spreads the IO requests over the full duration of the column access.

## System

We incorporate HPCache in Proteus [13, 23, 33], a pipelined analytical engine that uses LLVM-based code generation.

Proteus uses HetExchange [13] to parallelize query execution by injecting a set of metaoperators. Proteus represents queries as a set of pipelines. Specifically, HetExchange uses the router operator to parallelize execution: each router operator creates multiple instantiations of the pipeline above it and routes inputs to the different instances based on a routing policy. Pipeline instances are affinitized to specific processors, and execution across processors uses multiple pipeline instances. For example, a commonly used routing policy is a prefer-local policy. Under that policy, tasks are assigned to pipeline instances in the same NUMA region as the input data—and only fall back into assigning an input row group to a remote instance when there is skew. Further, routers do not perform any actual data transfers but only route tasks.

The mem-move operators handle data transfers. Such operators are often placed after routers and are responsible for fetching the data to local memory if they are not already there. For example, suppose a router decides, due to skew, to send a row group to a remote pipeline instance and a mem-move follows the router. In that case, the mem-move will transfer the corresponding data pages to the NUMA node local to the pipeline instance. The mem-move is also responsible for overlapping data transfers with the execution that follows it. In that regard, each mem-move operator instance has a consumer and producer side. The producer side accepts page handles, starts an asynchronous data transfer, and registers the transfer into a queue of pending transfers. The consumer side waits on that queue for completed transfers, and when a transfer completes, it removes it from the queue and pushes the corresponding data and task to the next operator. Each mem-move supports having multiple on-the-fly data transfers.

To integrate HPCache in Proteus, we extend the scan, router policies, and mem-move operators. When executing over storage-resident data, the scan operator emits page IDs instead of in-memory block handles. Further, we augmented the routing policies to be able to consider the NVMe location when deciding where to route a row group. Specifically, locality-based routing policies select the target pipeline instance based on the current location of the in-memory input data. For HPCache support, we extended the policies so that 1) if the data are not available in memory, they consider their location as the NUMA node closer to the NVMe holding the corresponding page, 2) if the data is cached in memory, the routing policy uses the location of the in-memory copy as the page’s current NUMA affinity.

Lastly, HPCache plugs into the mem-move operator to implement the NVMe-to-memory data transfers. Specifically, when the producer side of mem-move receives a page ID instead of an in-memory block handle, it will make a request from the buffer cache through the willNeedPage interface. The consumer side will do a getPage to wait for the transfer initiated by willNeedPage to complete. Multiple requests (transfers triggered by willNeedPage) will be on the fly, even from a single operator, which assists with hiding the access latency. If the page was already in the buffer cache, then willNeedPage wouldn’t trigger any storage reads, and getPage would return the page immediately. The rest of the operators are oblivious to the storage layer.

In Proteus, the page ID uniquely identifies the page and encodes the row group ID. Using the page ID, HPCache tracks the per-thread pipeline statistics in a simple thread private data structure through the calls to getPage. All pages within a row group are requested by the same pipeline instance, and each thread executes a single pipeline instance at a time. This data structure stores the time since the first page in the row group was requested, the columns accessed, the sum of times between the first page request for one row group, the first page request for the next, and the total count of requested row groups. As for fixed-width binary columnar data Proteus sizes each data page to be close to an OS huge page (exact size depends on the types in the rowgroup), HPCache has to store the above information once per few MBs of data. When the background thread invokes the optimization problem, it aggregates the per-thread statistics in order to calculate the inputs for the optimization problem. The background thread stores a history of these aggregated statistics at least as long as the look-back window (Sect. 4.3).

## Evaluation

In this section, we evaluate HPCache’s ability to efficiently use the available buffer cache, through a series of micro- and macro-experiments.

**Experimental setup.** Sect. 7.1 demonstrates the impact of varying both cached column proportions and storage bandwidth on query execution times through a set of micro-benchmarks. Further, we evaluate the accuracy and overhead of HPCache’s query execution time predictions as well as compare the exponential and fixed lookback formulations of the optimization problem.

In Sect. 7.2, we compare HPCache with an LRU eviction policy with different cache sizes and storage bandwidths. For workloads that involve many large scans, LRU is comparable to other policies that prioritize caching recently used pages.

**Hardware.** We use a dual-socket AMD EPYC 7413 server with Ubuntu 20.04 and kernel 5.4.0. Each CPU socket has 12 Corsair MP600 Pro NVMe drives, each using 4 PCIe 4.0 lanes, 256 GB of DRAM, and 24 cores. On each socket, we observe a maximum memory bandwidth of 116 GB/s per socket on the STREAM triad benchmark [27], and 84 GB/s sequential read bandwidth from the corresponding 12 socket-local NVMe drives, using fio [4]. In experiments that vary the storage bandwidth, we vary the number of NVMe drives and stripe the data across the selected drives.

**Software.** For all IO, we use io_ring with the O_DIRECT flag to bypass the operating system buffer cache. Our buffer cache uses 2 MiB pages to match the 2 MiB hugepages used by Proteus. We use the CVXPY [2, 16] library to solve the optimization problem outlined in Sect. 4.3.

### Micro-benchmarks

**Fig. 5:**
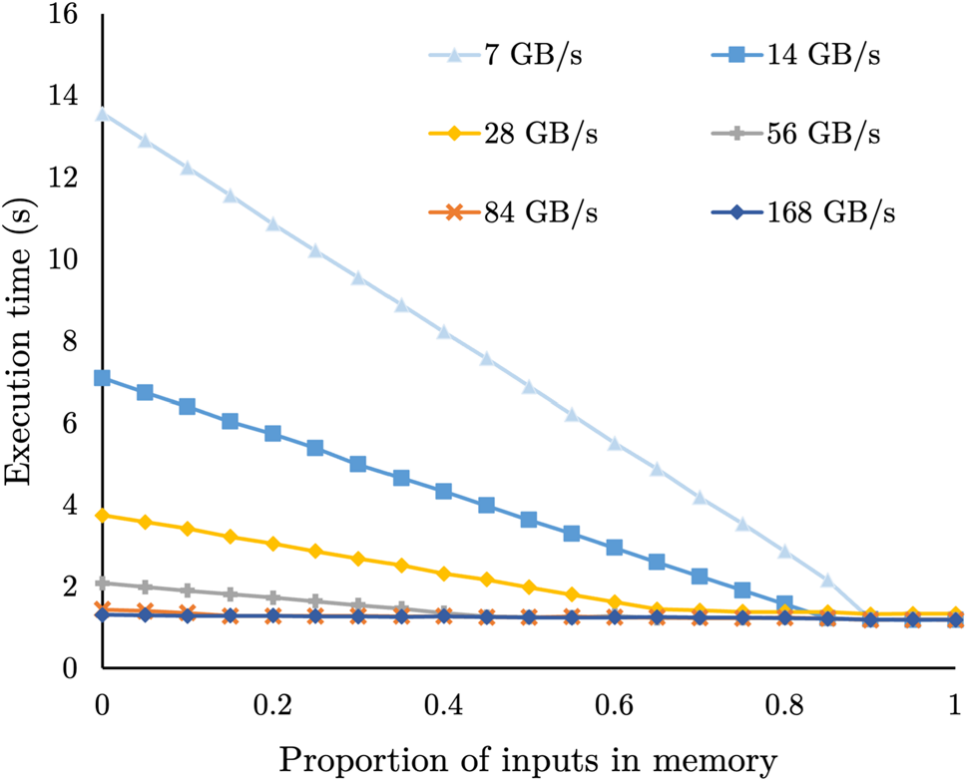
Execution time of SSB Q2.2 as the percentage of the queries inputs in memory range from 0% to 100%. Each series plots the execution time with a different storage bandwidth

**Proportional caching.** Fig. 5 evaluates the relationship between storage bandwidth and the amount of input data that need to be cached in memory to achieve peak query performance. We run SSB Q2.2 and vary the percentage of the input columns that are memory resident before the query begins from 0% to 100%. When data is fully loaded in memory, this query has a throughput of 80 GB/s, which is between the bandwidth of using 8 and 12 drives in our experimental setup. There is a linear decrease in execution time as the proportion of data in memory increases when the storage bandwidth is below 84 GB/s. When the storage bandwidth exceeds 84 GB/s, there is no benefit to caching data in memory as the query is no longer bottlenecked by storage bandwidth. As the storage bandwidth increases, the proportion of data that needs to be in memory before achieving the minimum execution time decreases. Further, once storage bandwidth exceeds the query throughput, there is no benefit to caching any data in memory.

**Fig. 6:**
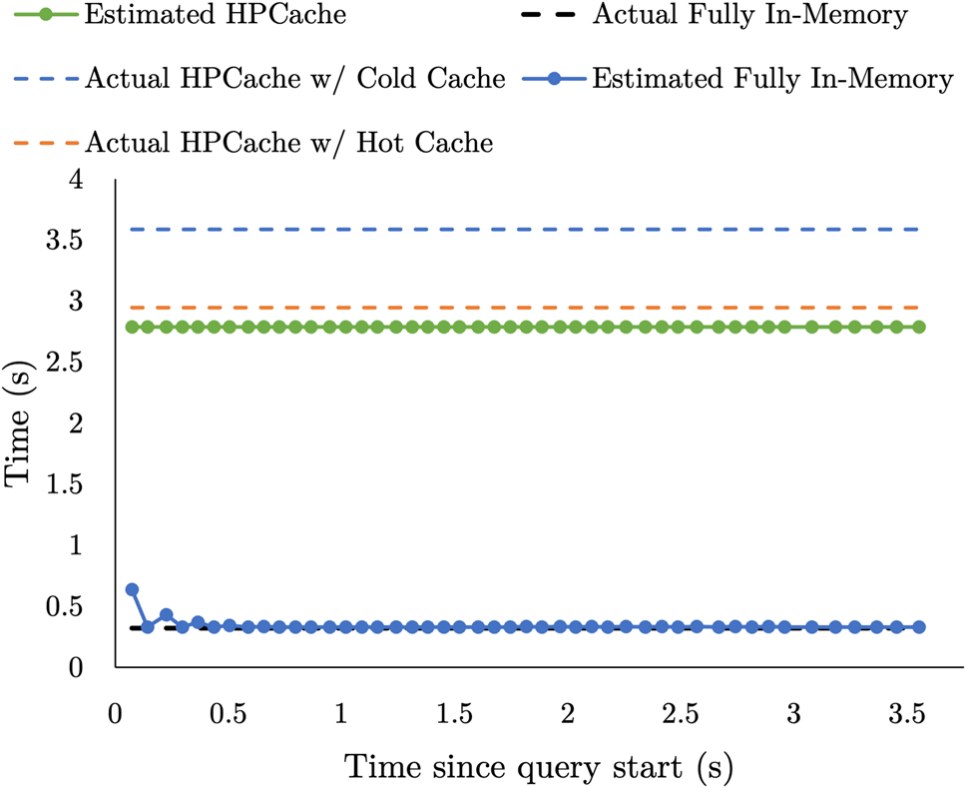
Runtime query execution time predictions vs actual execution times for SSB Q1.3

**Fig. 7:**
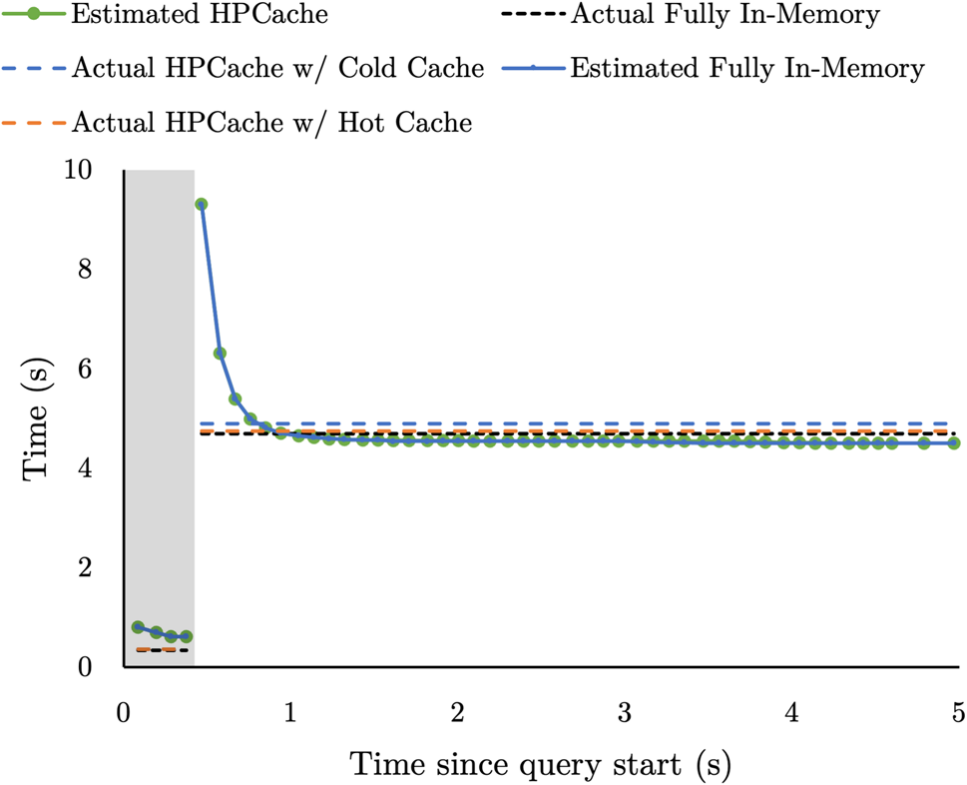
Runtime query execution time predictions vs actual execution times for SSB Q3.1

**Predicted execution times.** Fig. 6 and Fig. 7 evaluate the accuracy of the run-time estimates of the pipeline execution times for SSB Q1.3 and SSB Q3.1, respectively. We use SSB Q1.3 (a data-intensive query), and SSB Q3.1 (a processing-intensive query) to evaluate our model on both data-intensive and processing-intensive queries. Each query is run twice, starting from a cold cache with a cache budget of 25 GB, and HPCache evaluates the optimization problem every 100 ms.—approximately $^1/_4$ of its working set. SSB Q1.3 has two query pipelines. The first, shorter pipeline selects from the dimension table to build a hashtable for a hash join, and the second selects from the fact table and probes the hashtable. The build pipeline only consumes 4 pages of data and completes too quickly for HPCache to estimate execution times, so HPCache does not attempt to optimize that pipeline’s inputs. Hence, for Q1.3, the predictions are solely for the longer-running probe pipeline. SSB Q3.1 is similar; it has two small build pipelines (each executing in under 0.06 s), one slightly larger build pipeline and the long-running probe pipeline; HPCache makes estimates for the latter two pipelines; We plot the predicted pipeline execution times (per Eq. 1) made in the first execution of the query (which populates the cache), as well as the execution time of pipelines in the second execution with a hot cache. For SSB Q3.1, the shaded region in Fig. 7 marks the start of the first pipeline. The execution times of the queries when the input data is fully in memory are shown in the dashed black lines.

**Fig. 8:**
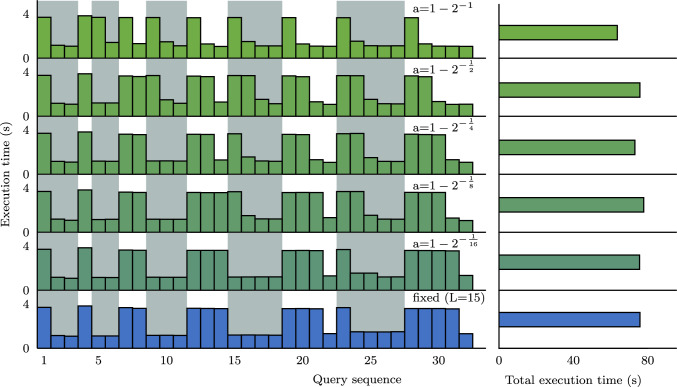
Caching impact when using a fixed look-back window (blue) versus exponential smoothing (green) versus, for various smoothing factors. Left: execution time per query (white background for query template 1, gray for query template 2). Right: total execution time across the full sequence

HPCache accurately models the in-memory execution times. However, early estimates in query pipeline execution do overestimate the in-memory execution times. In Proteus, parallelism is controlled by creating multiple instances of same the pipeline. When execution begins, there is some variation on when each pipeline instance begins consuming data. Since the system assumes all pipeline instances have equal throughput, this leads to the observed early overestimates. Since the Q1.3 probe pipeline is a data-intensive pipeline, the predicted HPCache time is based on the storage and memory bandwidth. In contrast to Q1.3, both pipelines shown for Q3.1 are processing-intensive, so the current run-time prediction and the in-memory prediction are identical and are both based on the pipeline’s predicted maximum throughput. The prediction model consistently underestimates the real execution time for both hot cache and in-memory execution. This is because the model assumes that all logical cores are only processing the pipeline. It does not account for anything else utilizing the CPU, such as the background prediction thread.

**Horizon effect and exponential smoothing.** Next, we show the issues that arise when using a fixed-length query window and how using the proposed exponential smoothing improves the cache efficiency.

*Methodology.* HPCache has access to 28 GB/s of storage bandwidth and 80 GB of memory for caching. The sequence begins with three iterations of Q1 and then alternates between increasing iterations of Q1 and Q2. The beginning and end of each sub-sequence of query template iterations are marked with a vertical dashed line. Both query templates have similar processing throughput exceeding the storage bandwidth and distinct inputs of equal size. They require caching in excess of the maximum cache size to approach in-memory performance. The optimization problem is evaluated every 200 ms.

*The fixed-horizon issue.* Fig. 8 plots the execution time for each query in this sequence on the left and the cumulative execution time of the sequence on the right. The sequence in blue at the bottom of the figure uses HPCache with a fixed-length window of 15 past queries to decide the column proportions—similar to the previous version of HPCache [30]. This fixed-window, linear formulation treats each query in the history window with equal weight. So HPCache minimizes the execution time of whichever query template appeared most often in the window of past queries. This can result in past query patterns inducing large changes in the column proportions as they drop out of the window. This effect can be seen in this plot, where the model is slow to adapt to Q2. It begins caching pages with Q2 at the 21st query in the sequence, and the impact of this is seen in the next query. This change occurs here because at query 21, there are eight instances of Q2 and seven of Q1 in the history window, so the perceived benefit of caching Q2 is finally greater than the benefit of caching for Q1. At every point earlier in the sequence, there are more instances of Q1 than Q2. The linear formulation can outperform when new queries appear for a short time before the sequence returns to the previous pattern, for example, at query 4, or queries 7 and 8 (where, in contrast, some exponential smoothing configurations are penalized in query 5 and 9 for changing their configuration).

**Fig. 9:**
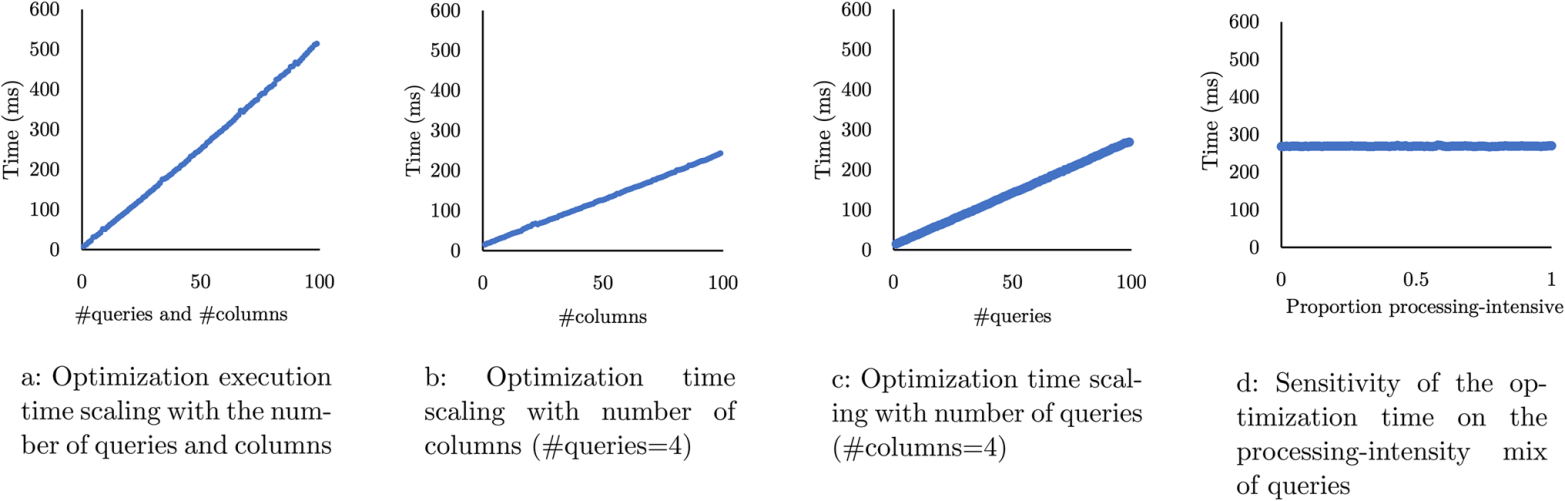
Scalability of the optimization problem

*Exponential smoothing to avoid horizon effects.* The green sequences in Fig. 8 show the execution times of the same sequence but using the exponential smoothing proposed in Sect. 4.4, which assigns higher weighs to more recent queries, and smoothly lowers the weight of past queries. In the green sequences, the topmost has the most aggressive value of the tuning parameter *a*, and the bottom has the least aggressive. For example, $a=1-2^{-1}$ gives greater weight to the execution time of the current and most recent queries. For this value of *a*, HPCache changes the cached column proportions at the beginning of each sub-sequence except for the first at query 4, where the weighted times of the previous four executions of Q1 exceed the weighted time of the current Q2. With less aggressive values of *a*, HPCache is gradually slower to change the cached column proportions. This can be seen in the figure when the query pattern shifts from Q1 to Q2 at query 22 (and back again at Q28). The least aggressive value ($a=1-2^{^{-1}/_{16}}$) behaves similarly to the equal weighting. Exponential smoothing reacts more quickly more quickly to changes in the query pattern. In this workload, this results in improved end-to-end performance as after query 4, each query is run at least twice in a row. Overall, using the aggressive exponential smoothing enables HPCache to achieve an additional 1.12x speed-up compared to the linear window.

**Fig. 10:**
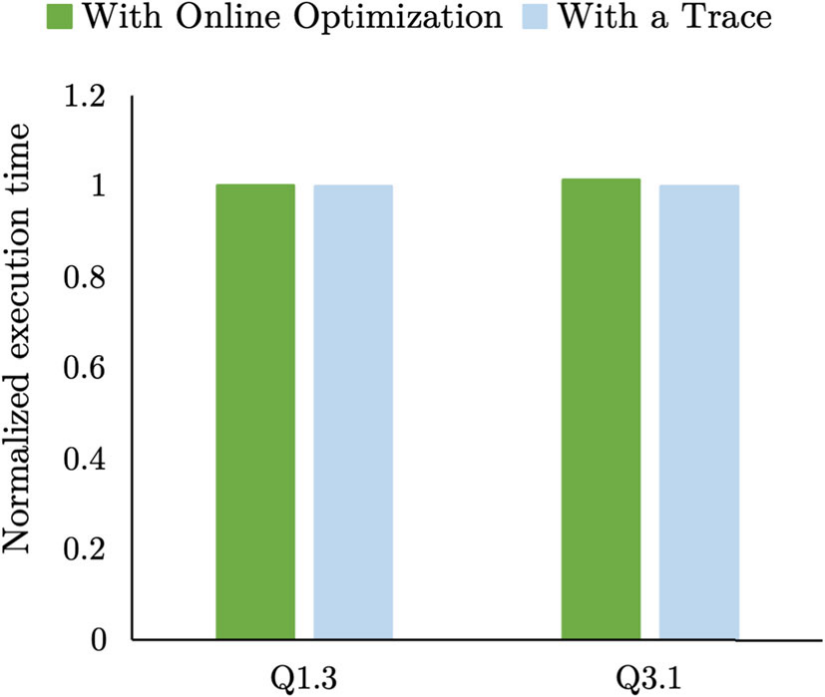
The overhead on query execution time from solving the optimization problem during query execution relative to using a trace of the outputs of the optimization problem. Execution times are normalized to the execution time when running with the trace. A 40GB cache and 4 NVMe drives are used

Optimization Overhead Fig. 9 shows how the optimization time scales with the number of columns and queries. Here, only the optimization problem run in order to isolate the optimization time. In Fig. 9a, we vary the number of distinct columns and the number of queries over those columns supplied to the optimization problem. Each query is data intensive, having a throughput greater than the storage bandwidth, and uses four random columns. For example, at point 40 on the x-axis, there are 40 distinct columns and 40 queries over those columns. The optimization problem scales linearly with both the number of queries and the number of distinct columns. Figure 9b and Fig. 9c show the same trend, but for the case where we keep the number of queries and columns, respectively, fixed at 4. The queries for both are again all data intensive. Fig. 9d plots the optimization time as the ratio of processing-intensive to data-intensive queries increases. 100 queries and 4 columns are used. Here, we can see that the optimization time is empirically independent of the query throughput. The optimization problem is sufficiently fast for the workloads discussed in this section. Note that even at 100 distinct columns, the HPCache’s optimization time is faster than the execution time of the simpler queries shown in the above experiments.

**Fig. 11:**
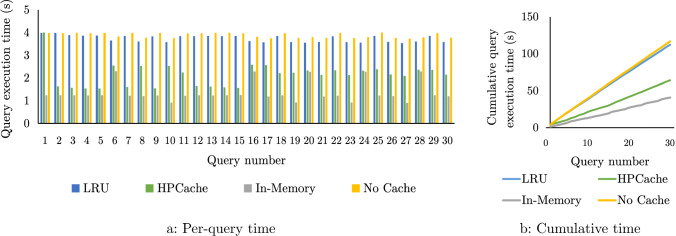
Per query and cumulative execution time of the query sequence using 80GB cache, which is smaller than the working set of one query. The data is striped across 4 drives (28 GB/s storage bandwidth)

Fig. 10 shows the impact on query execution times of solving the optimization problem during query execution. SSB queries Q1.3 and Q3.1 are run using a hot cache. The green bars show HPCache, which is solving the optimization problem every 200 ms. The blue bar shows a modified HPCache. The modified HPCache uses a trace file of the outputs of the optimization problem to mock the call to solve the optimization problem. Due to the very small absolute differences, the queries are run 50 times each, and we report the average execution times. For the data-intensive Q1.3, the difference is 0.3% and nearly indistinguishable from measurement noise. For the processing-intensive Q3.1, the difference is 1.4%. The overhead on query execution time of solving the optimization problem is relatively low. For longer queries, it would be feasible to further reduce the overhead by not resolving the problem once the outputs are stable, but we chose not to in our implementation.

**Fig. 12:**
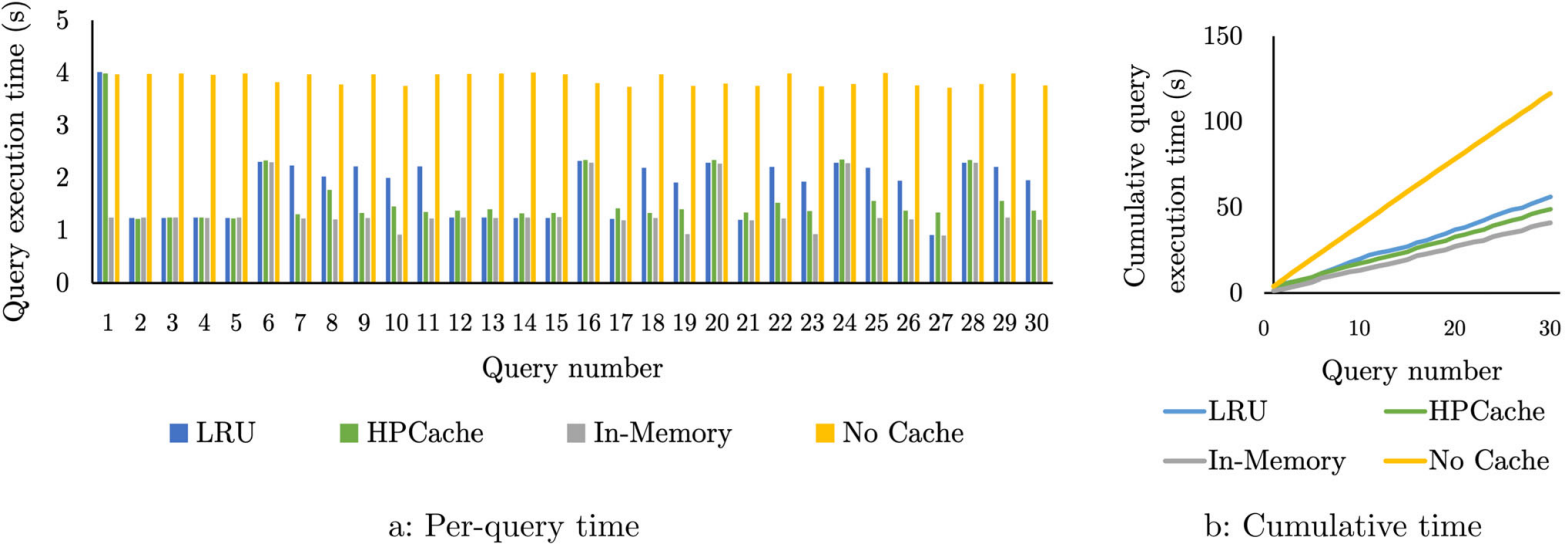
Per query and cumulative execution time of the query sequence using 120GB cache, which is smaller than the working set of one query. The data are striped across 4 drives (28 GB/s storage bandwidth)

### End-to-end evaluation

In this subsection, we evaluate HPCache’s end-to-end performance on a sequence of queries, demonstrating its effectiveness for varying cache sizes and storage bandwidths.

Methodology We evaluate on a sequence of 30 random queries from 4 query templates (QC and SSB Q2.1, Q2.2 and Q2.3) on the Star Schema Benchmark (SSB) data at a scale factor of 1000 [32]. QC joins the lineorder table with the customer and date tables and then groups by customer. Two of the four input lineorder columns of QC overlap with the other queries. Each query template has four input columns totaling 96 GB of inputs. All caching policies are evaluated starting from an empty cache. The system is warmed up by executing one query, and then, the caches are dropped before running the query sequence. To run in-memory, we use a caching policy that never evicts, and the cache is pre-populated with the full working set. To run without caching, we use a caching policy that immediately evicts each page once it has been consumed by the requesting query. The least recently used (LRU) policy is implemented using the standard hash table and linked list approach to track the most recently used pages. Further, our LRU policy is partitioned to reduce the mutex contention for the updates necessary to the linked list and hash table on each page request. The in-memory, non-caching, and LRU cache policies do not track the per-query or per-column statistics outlined in Sect. 5.3, but LRU does track the per-page recency of access.

**Avoiding cache thrashing.** In the next experiments, we evaluate HPCache’s ability to avoid cache thrashing and compare it with prior approaches. *Thrashing: bigger-than-cache working sets.* Fig. 11 plots the per query and cumulative execution times of the query sequence with 28 GB/s of storage bandwidth and an 80 GB cache, which is smaller than the working set of any one query. LRU suffers from cache thrashing within the execution of a single query and, therefore, has performance comparable to not caching at all. For this workload, LRU is representative of policies that cache recently used pages. Since the workload is composed of queries performing column scans, other policies such as 2Q [22] designed to resist scan thrashing would behave similarly. In contrast, HPCache optimizes over a history of queries instead of hit rates and achieves a 1.75x speedup over LRU.

*Thrashing: smaller-than-cache working sets.* When the cache size is greater than one query’s working set, LRU can improve execution times for successive queries accessing the same data. In Fig. 12, the 120 GB cache size exceeds the working set of one query. For example, queries 1 through 5 access the same columns, and query 6 has only a two-column overlap with the prior queries. Thus, in the sub-sequence of queries 1-5, LRU does not suffer from intra-query thrashing, and so when two subsequent queries have overlapping working sets it performs better. However, on query 6, where there is partial overlap, but a query with lower processing throughput (shown by the slower in-memory execution time), LRU again suffers from cache-thrashing. On this query, HPCache achieves a 1.72x speedup. However, HPCache does not result in a speedup for every individual query. In the sub-sequence of queries 11-15 that share inputs, HPCache is faster for query 11 but slower for queries 12-15 as it does not fully cache these inputs. Overall, HPCache performs better on the workload through improved memory efficiency, achieving a speedup of 1.14x on the total execution time.

**Fig. 13:**
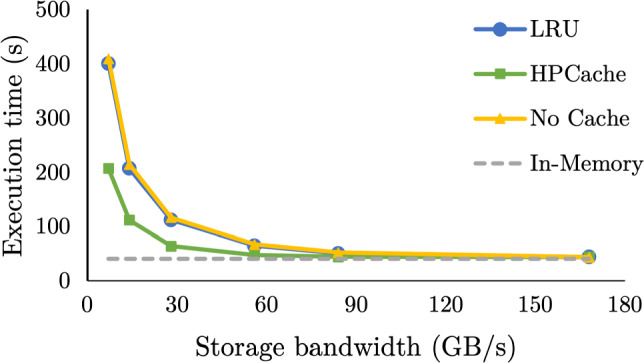
Total execution time of the query sequence in Fig. 11 as the storage bandwidth increases. (80GB cache)

**Fig. 14:**
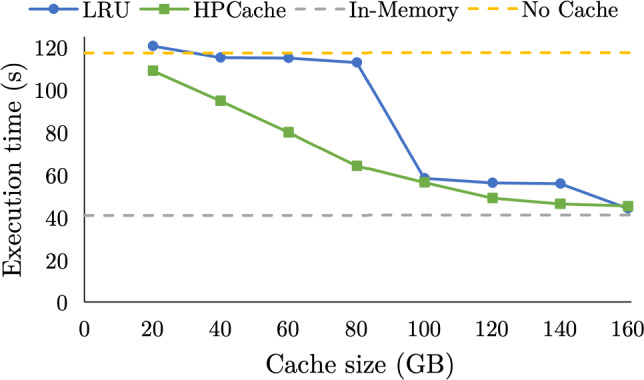
Total execution time for the query sequence using 4 drives and varying the cache size from 20GB to 160GB

**Effective caching when varying storage bandwidths.** Figure 13 shows the cumulative execution time for the same query sequence with an 80GB cache as the storage bandwidth varies. The execution time for the sequence when run fully in-memory and with no caching is also shown. In memory and no caching are the lower and upper bounds for execution time. As the storage bandwidth increases, the benefit of caching diminishes, and both policies converge to in-memory execution times. Though HPCache approaches full in-memory execution faster than LRU. At 56 GB/s HPCache is only 14% slower than memory, and at 84 GB/s just 7% slower. In comparison, LRU is 36% and 19% slower for the same respective storage bandwidths.

**Efficient cache utilization.** Fig. 14 shows the cumulative execution time for the query sequence using four drives (28 GB/s storage bandwidth) as the cache size is increased from 20 GB to 200 GB. At 20 GB, LRU performs marginally slower than no caching at all, as there are no cache hits and the overheads of maintaining the LRU data structures. In contrast, HPCache uses the 20 GB available memory to improve execution time by 8% relative to not caching. Between 20 GB and 80 GB HPCache improves steadily. LRU sees a slight improvement, as our partitioned LRU implementation means that there are some cache hits on subsequent queries with the same working set and when the cache size is a sizable fraction of a single query’s working set. There is a large jump in performance for LRU between 80 GB and 100 GB as with a 100 GB cache, LRU can fully cache the inputs of a query, so when two queries run back-to-back with the same working set, the page requests in the second query will hit the cache. LRU again plateaus between 100 GB and 140 GB until the entire working set of all the queries can be cached with a 160 GB cache. Both LRU and HPCache approach in-memory performance with a 160 GB cache but do not achieve in-memory performance as the data must first be loaded from storage the first time it is used in the sequence. Overall, HPCache achieves steady performance improvements across the full range of cache sizes, efficiently using the available memory.

## Background and related work

**OS-assisted buffer pools.** Due to the difficulty of implementing an efficient buffer pool, systems have often abandoned buffer pools altogether and instead relied on the operating system kernel’s paging capabilities, often through mmap; for example, MonetDB relies on memory-mapped IO to support larger than memory datasets [7, 20]. Memory-mapped IO offloads a significant part of the buffer pool implementation to the OS and offers hardware-assisted page-fault handling. However, memory-mapped IO also reduces the control that the database engines have over buffer management and can even result in sufficient contention and reduced prefetching that prevents the database engine from saturating NVMe arrays [14]. Vmcache is a buffer pool design that uses virtual memory but retains DBMS control over page eviction by using anonymous rather than file-backed virtual memory along with the MADV_DONTNEED hint, and addresses OS page table scalability issues through a kernel module [24].

**Optimizing buffer pool accesses.** Providing persistency and support for out-of-memory data traditionally introduces two overheads with respect to the buffer pool. First, having a centralized buffer pool creates a point of contention [21]. Second, persistency requires a level of indirection when translating in-memory references to out-of-memory object references. Graefe et al. [19] use pointer swizzling to eliminate buffer pool overheads when all data fits in memory. Pointer swizzling dereferences page references and replaces swizzled pointers with in-memory pointers. By avoiding a hashtable, they avoid a costly central point of contention. LeanStore [25] extends on pointer swizzling by speculatively unswizzling pages, which keeps hot pages in memory without explicitly tracking page accesses in shared data structure. Umbra [29] extends LeanStore with support for variable length buffer frames, improving handling of large objects. Overall, optimizing buffer pool accesses reduces the overhead imposed on mostly in-memory analytics while adding support for out-of-memory data. In contrast, HPCache improves the cache efficiency with respect to the performance gains achieved by caching data in memory.

**Buffer pool eviction policies.** Apart from handling larger-than-memory datasets, traditionally, buffer pools promise efficient in-memory data caching. Multiple eviction policies have been proposed to increase the cache hit frequency, using the databases’ access patterns, such as partitioning the buffer pool by relation [38], or into priority or access patterns zones and using an access pattern-optimized eviction policy inside each partition [31, 34]. Partitions are sized based on the expected performance benefit. Other approaches provide each query with a buffer large enough for the queries modeled hotset of pages [35]. LRU and MRU are used to increase the hit chance inside each partition, with second-chance eviction policies like 2Q reducing the cache pollution [22]. However, directly attached NVMe arrays provide significant bandwidth to make data scans competitive to query execution times. Instead of relying on frequency-based cache eviction, HPCache takes into consideration the overall benefit of data caching and prioritizes caching of high-benefit data over frequently loaded but low-benefit inputs.

**Heterogeneous storage.** The multitude of available storage devices provides a rich spectrum of performance and budget tradeoffs. Do et al. [17] reduce the computational cost of log-structured storage by offloading the computation required for garbage collection and recovery onto computational SSDs. Mosaic [39] is a storage engine specialized for scan-heavy workloads. It calculates performance-budget Pareto-optimal data placements for data residing across multiple types of storage devices. Mosaic uses workload traces to model column-granular data placement as an optimization problem and solves it offline using linear optimization. Borovica et al. [10] propose Skipper, an execution framework optimized for cold storage devices (CSD): as CSD can result in high delays when accessing data from powered-off disks, Skipper uses out-of-order execution to codesign the execution order and disk requests to hide unnecessary latencies. Both Skipper and Mosaic optimize the performance and cost of analytics on scan-heavy workloads, assuming the storage medium is the bottleneck. In contrast, in this work, we focus on improving analytical response times over high-bandwidth, directly attached NVMes.

## Conclusion

In this paper, we show that (i) caching pages that are accessed with the same frequency can yield significantly different query acceleration results, and (ii) optimizing the memory footprint requires partial column caching to avoid diminishing returns. We proposed HPCache, an eviction policy and tuning agent that optimizes the caching decisions of the buffer pool for analytical workloads on high-bandwidth storage. HPCache both inspects query execution to predict caching benefits and automatically tunes page caching priority. HPCache improves the efficiency of in-memory data caching for analytics, allowing faster query execution time and improved NVMe bandwidth utilization for a given memory budget. HPCache achieves up to a 1.75x speed up compared to an LRU eviction policy.

## Footnotes

1 The rest of this paper uses the term storage to refer to block storage such as hard disk drives (HDDs) and NVMe solid-state drives (SSDs).

2 We borrow the definition of pipeline from Neumann [28], where a pipeline is the sequence of operators between two fully blocking pipeline breakers or between an input and a fully blocking pipeline breaker. Fully blocking pipeline breakers are operators that have to consume their full input before producing any output (e.g., aggregation and join’s build side). Caching input data will only affect the throughput of a pipeline starting in an operator that accesses those inputs.

3 We use *x* both as a percentage, in the form 70%, in subscript, and as a proportion, in the form 0.7, within equations.
